# Guessing versus Choosing an Upcoming Task

**DOI:** 10.3389/fpsyg.2016.00396

**Published:** 2016-03-22

**Authors:** Thomas Kleinsorge, Juliane Scheil

**Affiliations:** Leibniz Research Centre for Working Environment and Human FactorsDortmund, Germany

**Keywords:** task switching, cognitive control, prediction, prediction error, predictive coding

## Abstract

We compared the effects of guessing vs. choosing an upcoming task. In a task-switching paradigm with four tasks, two groups of participants were asked to either guess or choose which task will be presented next under otherwise identical conditions. The upcoming task corresponded to participants’ guesses or choices in 75 % of the trials. However, only participants in the Choosing condition were correctly informed about this, whereas participants in the Guessing condition were told that tasks were determined at random. In the Guessing condition, we replicated previous findings of a pronounced reduction of switch costs in case of incorrect guesses. This switch cost reduction was considerably less pronounced with denied choices in the Choosing condition. We suggest that in the Choosing condition, the signaling of prediction errors associated with denied choices is attenuated because a certain proportion of denied choices is consistent with the overall representation of the situation as conveyed by task instructions. In the Guessing condition, in contrast, the mismatch of guessed and actual task is resolved solely on the level of individual trials by strengthening the representation of the actual task.

## Introduction

The human brain is an intrinsically predictive device. Predictive processes pervade probably all areas of cognition, ranging from short-term phenomena as perceiving a certain object in a particular position or reaching toward such an object to long-term decisions like planning a career or choosing a mate to share (at least large parts of) one’s life with (cf. Bubic et al., 2010, for a review).

Although notions of predictive processing can be found already in the writings of nineteenth century pioneers of psychology and neuroscience such as James and von Helmholtz, there is also a strong tradition within psychology, dating back to the days of behaviorism and being rooted in Cartesian conceptions of the mind, which construes behavior as being essentially stimulus-driven and reactive. And while the concept of a predictive brain has received overwhelming empirical support during the past decade, the legacy of behaviorism still lurks through many paradigms employed in experimental psychology as external stimulation is considered as the trigger for cognitive processes. This situation engenders the danger of mistakenly transferring the temporal structure of psychological experiments to the cognitive processes underlying the performance of an experimental task (cf. Kunde et al., 2007).

Correct predictions convey obvious adaptive benefits as they shorten the time needed for perceiving an expected stimulus and allow for preparing appropriate actions (LaBerge, 1995). Less obvious is the flipside of this, namely the consequences incurred by incorrect predictions. In the attentional domain, costs incurred by incorrect predictions (usually induced by misleading cues) are pervasive (Posner, 1980), as are costs associated with ‘re-programming’ a movement in the motor domain (Rosenbaum, 1985). However, whereas it may be considered as obvious (or even trivial) that correctly predicting that a particular event will occur conveys benefits as compared to a situation in which the prediction of an event is followed by the occurrence of another, unpredicted event, this obviousness may in part result from mistakenly transferring temporal structure from an experimental situation to inferred temporal structure of underlying cognitive and neural processes (cf. Mars et al., 2007). Predictions are usually embedded in overarching action plans so that costs on a lower level may convey benefits on a higher level by allowing for a fine-tuning of actions to actual demands. Furthermore, while a mismatch of predictions and reality may disrupt ongoing information processing and action, it subsequently promotes learning and other adaptations serving to enhance the efficiency of future behavior (cf. Bubic et al., 2010). A large body of evidence suggests that a key function of prediction errors is to induce hierarchically higher levels of information processing to adapt their predictions regarding the state of the world as conveyed by hierarchically lower levels, thus refining a model of the causal structure of the world by iteratively reducing the prediction error (cf. den Ouden et al., 2012; Clark, 2013).

In a recent study in the realm of task switching, we (Kleinsorge and Scheil, 2015) asked participants to predict the identity of the upcoming task (Experiments 1 and 2). Since the actual task was determined quasi-randomly, participants had to resort to guessing in order to meet the task requirements. We hypothesized that a mismatch of predictions with the actually presented task results in an additional recruitment of cognitive control processes, which in turn should facilitate task switches more than task repetitions. As a consequence, switch costs, i.e., an increase in reaction times (RTs) and error rates (ERs) in task switch trials as compared to task repetition trials, should be less pronounced after an incorrect as compared to a correct guess of the upcoming task. The latter prediction was borne out by the data. However, this finding was mainly due to an increase of task-repetition RTs following an incorrect guess. We accounted for these observations by assuming that incorrect guesses would generally interrupt ongoing information processing but that in task switch trials, this would be offset by a transient boost of cognitive control selectively facilitating task switches. When, in a third experiment, participants were asked to guess the position of the next precue instead of the identity of the next task, we observed a selective facilitation of task switches in case of an incorrect guess. A similar finding had been reported by Duthoo et al. (2012).

In the present study, we replicated the basic procedure of Experiment 2 of Kleinsorge and Scheil (2015) with the addition of another, newly designed Choosing condition. The two conditions, Guessing and Choosing, were strictly identical with the exception of the instructions given to participants. Therefore, any performance differences between these two conditions could not be due to differences in stimuli, responses, or the tasks and their sequence. Importantly, both conditions differed from the original experiment of Kleinsorge and Scheil (2015) in that the upcoming task conformed to participants’ guesses or choices in 75 percent of the trials. Whereas participants in the Choosing condition were correctly informed about this, participants in the Guessing conditions were told that the upcoming task was determined independently of their guessing response. The rationale of the experiment hinged on the expectation that this change would not affect participants performance as evidenced by a replication of the interaction of the ‘correctness’ of the guessing response and the task sequence, that is, whether the task repeated or switched. Fortunately, our observations conformed to this expectation (see below). The proportion of 75% of trials conforming to participants’ guesses or choices was chosen because we anticipated that this proportion would lend sufficient credibility to both instructions. Increasing the number of ‘correct guesses’ would have made it increasingly likely that participants experience control over the presented task, whereas decreasing the number of fulfilled choices would have counteracted the experience of control that is essential for an experience of ‘choice.’

Of course, the Guessing and Choosing conditions differed not only with respect to what participants were instructed to do (to guess vs. to choose) but also with respect to the amount and validity of information provided to them. We consider these differences not as a confound but as corollaries of the different mental states associated with guessing and choosing: ‘to guess’ means that one experiences no control over a situation (even if one in fact may have some), whereas to choose is intrinsically associated with experiencing at least some control (even if one may actually have none). Nevertheless, this implies some ambiguity with respect to attributing eventual differences between conditions to different instructions and different amounts of information provided to participants.

As far as we can see, there is little current knowledge for guiding specific hypotheses regarding functional differences between guessing and choosing under otherwise strictly identical conditions. Bode et al. (2013) reported a functional magnetic resonance imaging (fMRI) study in which participants were asked to guess or decide (choose) among object categories without any perceptual information supporting their guesses or choices. Based on a fMRI-based pattern classifier, they were able to cross-predict choices from decisions and vice versa. The authors interpreted their findings as indicating that (perceptual) guesses and decisions were partly based on the same neural mechanisms, which they located mainly in the mid-precuneus, a structure located in medial parietal cortex that is assumed to be involved in self-related processing and the experience of agency (Cavanna and Trimble, 2006).

While, as shown in our previous study (Kleinsorge and Scheil, 2015), incorrect guesses of an upcoming task certainly result in some kind of adaptation, there is no information available to participants allowing for an enhancement of the accuracy of future guesses. At the same time, the uncertainty inherent in the unpredictability of the environment should motivate the cognitive system to ‘try harder’ in order to attain some control over the situation. In the Choosing condition of the present study, in contrast, participants were veridically informed about the underlying probabilities. This provided participants with an internal representation or model of the situation which could be compared to the actually experienced frequencies of granted and denied choices. Because these frequencies should match the internal model based on instructions, granted as well as denied choices may corroborate rather than challenge participants’ current model of the situation without any need for adjustments on a higher level. From this perspective, one may expect that while denied choices should result in some delay of responding due to the necessity to update the representation of task identity, they should not induce any adaptations that affect task repetitions and switches differently. Observing such a difference between the Guessing and the Choosing conditions would result in two advances. First, it would corroborate our assumption that what we observed in our previous study (and sought to replicate in the Guessing condition of the present study) was not due to any confounds related to the task sequence such as different probabilities of task repetitions and task switches or disruptions of repetition-based facilitation that affected incorrectly guessed task repetitions (cf. Kleinsorge and Scheil, 2015, for a discussion) because all these factors should affect the Guessing and Choosing conditions in the same way. Second, it would provide conclusive evidence that incorrect guesses indeed trigger a top-down process of cognitive control.

## Materials and Methods

### Participants

Thirty-eight subjects (11 male) with a mean age of 23.8 years (range: 20–29) participated. All had normal or corrected-to-normal vision. They were assigned to one of the two groups based on an odd-even scheme. The study was approved by the local ethics committee of the Leibniz Research Centre for Working Environment and Human Factors. All participants gave their written informed consent for study participation.

### Stimuli, Tasks, and Apparatus

Imperative stimuli consisted of digits from range 1–9 (excluding 5) and the letters A, B, G, E, N, O, S, and U. Each digit was about 7 mm high × 4 mm wide. Digits and letters were presented side by side, their position was chosen randomly on every trial. Task precues consisted of a dark blue square, diamond, circle, or triangle surrounding the position of the imperative stimulus with a size of about 15 cm × 15 cm. There were four tasks in total, two of them regarding the digit and two regarding the letter. The numerical judgment tasks either concerned the magnitude (smaller vs. larger than five) or the parity of the digits. The magnitude task was indicated by the diamond, the parity task was indicated by the circle. The letters had to be judged regarding their position in the alphabet (first or second half, indicated by the triangle) or regarding whether it is a consonant or a vowel (indicated by the square).

Stimuli were presented centrally on a 17″ monitor in black on light-gray background. Viewing distance was not controlled, but equally given with approximately 60 cm. Responses were made with the right hand by pressing the left arrow key for small and even digits as well as for vowels and letters from the first half of the alphabet. The right arrow key had to be pressed for large and odd digits and for consonants and letters from the second half of the alphabet.

### Design and Procedure

The experiment consisted of three phases. The first and third phase were designed as the usual cuing-variant of the task switching paradigm, with a task switching probability of 0.5. During the second phase, participants determined the upcoming task in 75% of the trials, whereas a different task was presented in 25%. The only difference between the two groups were the instructions they received at the beginning of the second phase, whereas the experimental procedure was completely identical for both groups.

At the beginning of the experiment, participants were provided with on-screen instructions in which the tasks and the meaning of the task cues were explained. The first phase consisted of three blocks of 96 trials. The response-stimulus interval (RSI) was set to 1,100 ms in the first and third phase of the experiment and to 3,100 ms during the second (prediction) phase. In case of an error, error feedback was presented for additional 1,000 ms; in case of RTs slower than the RT deadline of 2,500 ms, RT feedback (‘too slow’) was presented for additional 1,000 ms. Two CTIs of 200 and 1,000 ms were employed during the whole experiment, with the duration of the CTI being evenly and pseudo-randomly distributed across all tasks.

At the beginning of the second phase, participants of the two groups received different instructions. Participants of the Choosing condition were informed that during this phase, they could choose the upcoming task in every trial. This was done by pressing one of four keys on a custom-built keyboard with the left index finger. During the first 2,000 ms of the RSI, a choosing request (‘Please choose now’) was presented on the screen. Within this time, participants had to press one of the choosing keys and to hold it down until the presentation of the imperative stimulus. If the key was dropped earlier, error feedback was presented and the trial was canceled. If the choosing key was pressed after 2,000 ms, participants received feedback (‘Too slow! Please choose faster’) until one of the keys was pressed, but the trial was not canceled. Participants were informed that in 75% of the trials, the specified task would be presented (accepted choices), but in 25%, another task would be presented at random (denied choices). They were instructed to choose all tasks approximately equally often and with a switching probability of 0.5. After choosing, the trial continued as during the first phase: first, a fixation mark and after that, the precue was presented. Fixation mark and precue summed up to 1,100 ms, the CTIs being the same as in the first phase. After that, the imperative stimulus was presented and had to be judged regarding the task that was indicated by the precue.

Participants of the Guessing group were told that the task sequence during the next blocks would be completely random, as it was in the first phase. They were instructed to guess on every trial which task will have to be performed on next. The guessing procedure was identical to the choosing procedure, except for a different wording of the guessing request (‘Please guess now’) and the feedback for slow guesses (‘Too slow! Please guess faster’). Participants were told that the task sequence was completely random, that no regularity had to be ‘detected’ and that they were expected to really guess the next task. In addition, they were told that all tasks would occur with equal frequency and that switching probability was 0.5. The second phase consisted of nine blocks of 96 trials each.

At the beginning of the third phase, participants were informed that, as in phase 1, no choosing/guessing was required. The third phase consisted of three blocks of 96 trials each.

## Results

The data were analyzed by two sets of analyses of variance. The first set of analyses compared performance across all phases of the experiment. In these analyses, the effect of the CTI was taken as an indicator of preparation for a guessed or chosen task in advance of the presentation of the task precue. In a second step, the data from the second phase were analyzed separately. These analyses focused on the effects of task expectation and its interaction with the task sequence. Of special interest was the question inasmuch a difference between choosing and guessing would be observed in this respect.

### Analyses across All Phases of the Experiment

In a first step, mean individual RTs and ERs of all three phases of the experiment were analyzed as a function of the between-participants factor Condition (Guessing vs. Choosing) and the within-participants factors Phase (first, second, third), CTI (200 vs. 1,000 ms), and Task Transition (repetition vs. switch). From the second phase, only trials with correct guesses or granted choices were included into this analysis. The first blocks of the first and second phase were considered as practice and not included in the analyses. Trials in which the RT deadline was missed (1.8%) and trials following an error (9.8%) were also discarded, as were error trials from the analysis of RTs (6.9%). Note that errors in post-error trials were counted as post-error trials, which explains the higher percentage of these trials relative to error trials. The main aim of these analyses was to check inasmuch participants were committed to their guesses or choices in the second phase. Such a commitment should be indicated by a reduced effect of the CTI in the second as compared to the first and third phases of the experiment because participants could initiate preparation for the guessed or chosen task already in advance of the preparation of the task cue. Indeed, the reduction of RT induced by a prolongation of the CTI amounted to 190 and 167 ms in the first and third phase, respectively, but to only 105 ms in the second phase. This gave rise to a significant interaction of Phase and CTI, *F*(2,72) = 14.16, *MSe* = 5,148, *p* < 0.0001, on top of a main effect of CTI, *F*(1,36) = 359.86, *MSe* = 7,373, *p* < 0.0001. The main effect of Phase was also significant, *F*(2,72) = 69.28, *MSe* = 34,136, *p* < 0.0001, due to a monotonic decrease of mean RT from 1,182 over 1,066 to 901 ms from the first to the third Phase. Mean switch costs amounted to 192 ms, *F*(1,36) = 241.8, *MSe* = 16,938, *p* < 0.0001. Switch costs were largest in the first phase (304 ms), substantially declined in the second phase (119 ms), and became larger again in the third phase (153 ms), *F*(2,72) = 35.42, *MSe* = 10,223, *p* < 0.0001. Furthermore, switch costs were reduced from 265 ms with the short CTI to 120 ms with the long CTI, *F*(1,36) = 148,54, *MSe* = 3,924, *p* < 0.0001. This reduction of switch costs by a prolongation of the CTI differed across the three phases, *F*(2,72) = 4.58, *MSe* = 3,891, *p* < 0.05. In the first phase, switch costs were reduced from 389 to 220 ms. In the second phase, switch costs were reduced from 166 to 68 ms, and in the third phase, the reduction was from 238 to 68 ms. The only significant effect involving the between-participants factor Condition was the second-order interaction of Condition, CTI, and Task Transition, *F*(1,36) = 5.48, *MSe* = 3,924, *p* < 0.05. Whereas the magnitude of switch costs was roughly the same with a long CTI across the Guessing and Choosing conditions (118 vs. 121 ms), in the Choosing condition switch costs were smaller (238 ms) than in the Guessing condition (291 ms) with a short CTI.

The corresponding analysis of the ERs yielded a significant main effect of Phase, *F*(2,72) = 55.86, *MSe* = 0.014, *p* < 0.0001, with mean ER declining from 18% in the first phase to 5.1% in the second phase and 4.6% in the third phase. There was a main effect of CTI, *F*(1,36) = 11.88, *MSe* = 0.002, *p* < 0.01, being due to a reduction of ER from 9.8% with a short CTI to 8.4% with a long CTI. Mean error switch cost amounted to 4.4%, *F*(1,36) = 81.96, *MSe* = 0.003, *p* < 0.0001. The error switch cost declined across the three phases of the experiment (9.6, 2.2, and 1.4%), *F*(2,72) = 34.41, *MSe* = 0.002, *p* < 0.0001. The only effect involving the between-participants factor Condition was the interaction of Condition and Phase, *F*(2,72) = 3.23, *MSe* = 0.014, *p* < 0.05. In the Guessing condition, ERs declined from 14.3 to 4.7 and 4.5% in the first, second, and third phase. In the Choosing condition, the corresponding numbers amounted to 21.0, 5.5, and 4.7%. As the largest difference between the two conditions was observed in the first phase in which there was no difference between the Guessing and Choosing condition, the difference in ERs in the first phase can be considered as spurious. Regarding the seemingly steeper learning curve observed for the Choosing as compared to the Guessing condition, one may consider the larger amount of information provided in the Choosing condition as a possible explanation. However, this observation may also simply be due to uncontrolled between-participants variance. In any case, it is important to note that accuracy levels were comparable when our between-participants manipulation took effect.

### Analyses of the Second Phase

In the main analyses, individual mean RTs and ERs from the second phase of the experiment were analyzed as a function of the between-participants factor Condition (Guessing vs. Choosing) and the within-participants factors CTI (200 vs. 1,000 ms), Task Transition (repetition vs. switch), and Expectation (expected vs. unexpected). The exact meaning of the latter factor varied across conditions. In the Guessing condition, ‘expected’ means that the upcoming task corresponded to the guess of the participant, whereas ‘unexpected’ means that the actual task differed from the guessed one. In the Choosing condition, ‘expected’ corresponds to granted choices, whereas ‘unexpected’ corresponds to denied choices. The first block of the second phase was considered as practice and not included in the analyses. Trials in which the RT deadline was missed (0.4%) and trials following an error (8.7%) were also discarded, as were error trials from the analysis of RTs (6.2%).

The analysis of RTs (cf. **Table 1**) yielded significant main effects of CTI, *F*(1,36) = 247.06, *MSe* = 9,592, Task Transition, *F*(1,36) = 57.81, *MSe* = 10,203, and Expectation, *F*(1,36) = 46.28, *MSe* = 11,641, all *p*’s < 0.0001. A prolongation of the CTI reduced mean RT from 1101 to 922 ms. Task switches were associated with longer RTs (1,056 ms) than task repetitions (967 ms). Expected tasks went along with faster responses (969 ms) than unexpected tasks (1,054 ms). A prolongation of the CTI reduced mean switch costs from 119 ms to 60 ms, *F*(1,36) = 30.2, *MSe* = 2,166, *p* < 0.0001. A prolongation of the CTI also reduced the difference between expected and unexpected tasks from 159 to 11 ms, *F*(1,36) = 115.03, *MSe* = 3,490, *p* < 0.0001.

**Table 1:** Mean reaction times (RT) as a function of Condition, Expectation, Task Transition, and CTI (SEM in parentheses).

		Expected		Unexpected		*M*
		Task repetition	Task switch	Task repetition	Task switch	
Guessing	CTI 200 ms	958 (40)	1148 (38)	1158 (45)	1216 (49)	1120 (79)
Condition	CTI 1000 ms	903 (39)	978 (40)	933 (52)	963 (45)	944 (82)
Choosing	CTI 200 ms	919 (31)	1060 (33)	1130 (39)	1216 (42)	1081 (67)
Condition	CTI 1000 ms	857 (33)	926 (34)	875 (45)	940 (38)	899 (70)

*M*		909 (34)	1028 (35)	1024 (43)	1084 (42)	

**FIGURE 1:**
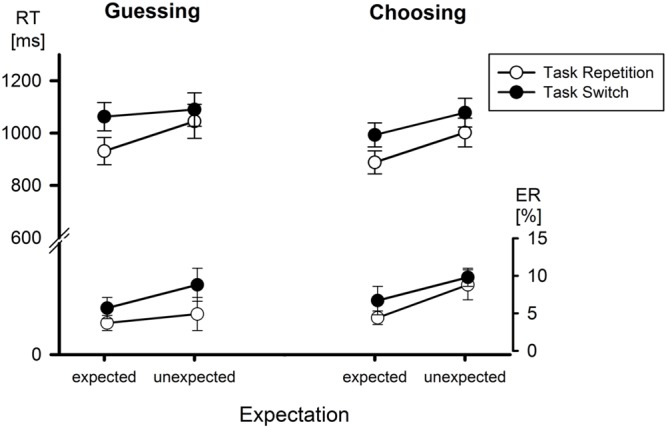
**Mean reaction times (RT) and error rates (ER) as a function of Condition, Expectation, and Task Transition.** Error bars represent SEM.

Of central importance is the interaction of Task Transition and Expectation, *F*(1,36) = 16.59, *p* < 0.001, and its modulation by Condition, *F*(1,36) = 4.19, *p* < 0.05, *MSe* = 3,821. In case of a task repetition, expected tasks went along with a mean RT of 909 ms, which was increased to 1,024 ms with an unexpected task. In case of a task switch, the increase of RT by an unexpected task was less pronounced (1,028 vs. 1,084 ms). In terms of switch costs, these amounted to 119 ms with expected tasks and were reduced to 60 ms with unexpected tasks. However, as can be seen from **Figure 1**, this interaction was more pronounced in the Guessing condition in which the switch cost reduction by task unexpectedness was from 130 to 45 ms, whereas the corresponding reduction in the Choosing condition was only from 105 to 76 ms. Besides, the second-level interaction of CTI, Task Transition, and Expectation turned out to be significant, *F*(1,36) = 4.62, *MSe* = 4,860, *p* < 0.05. With a short CTI, an unexpected task reduced switch costs from 166 to 72 ms as compared to an expected task. This reduction was less pronounced with a long CTI (72 vs. 47 ms).

The results of the corresponding analysis of ERs were consistent with those of the RT analysis. A prolongation of the CTI reduced mean ER from 7.4 to 5.3%, *F*(1,36) = 20.94, *MSe* = 0.0017, *p* < 0.0001. Compared to a task repetition (5.4%), ER was increased by a task switch to 7.3%, *F*(1,36) = 14.02, *MSe* = 0.0019, *p* < 0.001. Expected tasks were associated with a mean ER of 5.1%, which was increased to 7.6% in case of an unexpected task, *F*(1,36) = 12.77, *MSe* = 0.0037, *p* < 0.01. The cost associated with an unexpected task as compared to an expected task was more pronounced with a short (3.3%) as compared with a long (1.7%) CTI, *F*(1,36) = 5.02, *MSe* = 0.0011, *p* < 0.05. The only effect involving the between-participants factor Condition was the three-way interaction with CTI and Expectation, *F*(1,36) = 4.31, *MSe* = 0.0011, *p* < 0.05. This interaction was due to the fact that the reduction of the costs associated with an unexpected task by a prolongation of the CTI was exclusively observed in the Choosing condition (5.3 vs. 2.1%), whereas these costs were identical among the two CTIs in the Guessing condition (1.3%).

### Auxiliary Analysis

In order to check whether our main finding regarding the modulation of the interaction of Task Transition and Expectation by Condition may have been brought about by differences in the frequencies of predicting a task repetition vs. switch, we first explored whether such differences existed between the Guessing and Choosing conditions. This was indeed the case [*t*(36) = -2.96, *p* < 0.01]: whereas participants of the Choosing condition chose task switches with a mean probability 0.75, the corresponding probability was 0.64 in the Guessing condition. In order to test whether this difference in bias could account for the observed differences with respect to the interaction of Task Transition and Expectation, we subdivided both groups by median splits based on each participant’s relative frequency of predicting a task switch. In the Guessing condition, participants below the median exhibited a mean probability of predicting a task switch of 0.53, those above the median of 0.76. In the Choosing condition, the respective probabilities were 0.71 and 0.81. Most importantly, this did not affect the observed differences between the two conditions regarding the interaction of Task Transition an Expectation, with all interactions involving the median-split based variable Probability of Switch Prediction being far from significant (all *p*’s > 0.15). Descriptively, in the Guessing condition the switch cost reduction by task unexpectedness was from 152 to 54 ms for those participants with a probability of predicting a task switch below the median, and from 113 to 34 ms for those participants with a probability of predicting a task switch above the median. In the Choosing condition, the switch cost reduction by task unexpectedness was from 100 to 82 ms for those participants with a probability of predicting a task switch below the median, and from 113 to 64 ms for those participants with a probability of predicting a task switch above the median. Thus, whereas there was some trend in the Choosing condition for a stronger switch cost reduction by task unexpectedness for those participants with a probability of predicting a task switch above the median, this difference was statistically not significant (*p* > 0.25). It is also remarkable that in the Guessing condition, the reduction of switch cost by task unexpectedness decreased from 98 to 79 ms with increasing probability of predicting a task switch, whereas in the Choosing condition it increased from 18 to 49 ms. Furthermore, when one compares the two subgroups with the most similar probability of predicting a task switch (i.e., those above the median in the Guessing Condition and those below the median in the Choosing condition), the difference regarding the task switch reduction by task unexpectedness is even slightly more pronounced (79 vs. 18 ms) than the one depicted in **Figure 1** (85 vs. 29 ms). Therefore, we safely conclude that differences in bias regarding predictions of task repetitions and switches cannot account for this effect.

## Discussion

One part of the present study, the Guessing condition, was a conceptual replication of Experiment 2 of Kleinsorge and Scheil (2015). The new experiment differed from the original one mainly with respect to the fact that the determination of the actual task was in part dependent on the guessing response of the participant. This procedural variation did not result in any substantial change in participants’ performance. It was still observed that the presentation of an unexpected task hampered the performance of task repetitions more than the performance of task switches, which resulted in a reduction of switch costs with unexpected as compared to expected tasks.

In contrast, in the newly designed Choosing condition task (un)expectedness affected task repetitions and task switches in a more even manner. This observation has two important implications. First, it shows that the switch cost reduction observed in the Guessing condition cannot be caused exclusively by features of the task sequence or the interruption of this sequence by an interpolated activation of another task in case of a ‘wrong’ guess. In particular, it may be argued that the impact of incorrect guesses on task repetitions may be due to a disruption of repetition-based facilitation (cf. Kleinsorge and Scheil, 2015, for an extensive discussion of this point). However, such an account should apply likewise to the Guessing and Choosing conditions. Besides, accounts in terms of disruption of repetition-based facilitation had been already questioned by an additional experiment (Experiment 3 of Kleinsorge and Scheil, 2015) in which participants did not guess the upcoming task but the position of a laterally presented precue. In this case, incorrectly guessing the precue position reduced switch costs almost exclusively by reducing the RT of task switches.

The second implication relates to functional differences between guessing and choosing under otherwise identical conditions. As outlined in the introduction, there is almost no empirical work comparing differences or commonalities of guessing and choosing under otherwise strictly identical conditions. The work of Bode et al. (2013) suggests that both processes have a large functional overlap in neural terms, although this was investigated with a widely different task (perceptual discrimination). Nevertheless, our observations corroborate the assumption of large functional overlap in behavioral terms because we obtained only a very limited number of significant interactions with the factor Condition. One of these interactions, the interaction of Condition, Expectation, and CTI, may be interpreted as indicating that participants of the Choosing condition were initially somewhat more committed to their choice than participants of the Guessing condition were committed to their guess, a difference that was leveled off with a long CTI. However, this interaction was only observed in the ERs and not mirrored by a corresponding pattern in RTs.

The second and certainly theoretically most important interaction involving Condition was the interaction of Condition, Expectation, and Task Transition (cf. **Figure 1**). In the introduction, we considered that the truthful instruction of the Choosing condition may provide participants with a global model of the overall situation in terms of the probabilities of granted and denied choices that were met by what participants actually experienced in the course of the experiment. Therefore, even an unexpected task, albeit generating a mismatch on the local level of individual trials, provided no challenge to this global model. In contrast, in the Guessing condition, participants were told that the task sequence would be random. Therefore, the only global model offered to participants alluded to a more or less diffuse notion of ‘randomness.’ It does not seem that the deviation from randomness that we introduced in the present experiment affected participants in a systematic manner because we observed essentially the same pattern of performance as in Experiment 2 of Kleinsorge and Scheil (2015) in which the task sequence was indeed (quasi-)random. Thus, there is no indication that participants in the Guessing condition could relate the frequencies of correct and incorrect guesses to expected frequencies. Therefore, there were only mismatches on the local level of individual trials but nothing to learn in relation to the overall structure of the situation. Therefore, the only option for the cognitive system to adapt to the mismatch of expected and actual task was to enhance the activation of the actual task, a process that facilitated task switches more than repetitions.

The latter considerations are consistent with a predictive coding perspective as outlined by Clark (2013). According to this framework, the cognitive system, as a predictive device, constantly predicts its own future states on lower levels of a cognitive hierarchy. When these expectations are not met, error signals are generated on the, respectively, lower levels to which these predictions apply. These error signals induce modifications of the predictions on the, respectively, higher level that gave rise to the prediction error, thereby reducing this error. The crucial difference between the Guessing and Choosing conditions of the present experiment may consist of the level of the hierarchy on which task-relevant predictions are generated. In the Guessing condition, predictions (in the rather abstract sense considered here) may apply only to the level of tasks of an individual trial, with error signals generated on this level serving to efficiently disambiguate the actual task. In the Choosing condition, in contrast, a hierarchically higher level representing relative probabilities across individual trials may come into play. Because predictions generated on this level should not be challenged by what is experienced, a coherent representation of the situation is fostered which may modulate the impact of prediction errors arising from lower levels of the hierarchy. This, in turn, would deprive the system of a stimulus for enhancing the level of controlled processing, with the consequence of less switch cost reduction induced by an unexpected task. Of course, this does not imply that conflicts on the level of individual trials did not play any role in the Choosing condition, but only that the impact of such conflicts was dampened in comparison to the Guessing condition.

At present, these considerations are admittedly rather speculative. In addition, they are based on a framework that, although intended to capture principles on all levels of a cognitive hierarchy (cf. den Ouden et al., 2012), received its empirical support mainly from studies investigating more elementary features of neural processing (cf. Clark, 2013). The major implication of the account delineated in the previous paragraph is that the impact of error signals generated on a certain level of the cognitive hierarchy should be modulated by whether expectations concerning the present situation are also generated on a hierarchically higher, more abstract level.

Some support for these considerations comes from studies investigating the effects of unexpected auditory stimuli on different hierarchical levels. Todorovic and de Lange (2012) dissociated the effects of repetition suppression, that is, the reduction of neural activity by repeating a stimulus, and expectation suppression, that is, the reduction of neural activity by an expected stimulus. Neural activity was measured by magnetoencephalography. Stimuli consisted of pairs of tones, with the identity of the first tone predicting the identity of the second tone with a probability of 0.75. Expectation suppression was stronger when the first tone validly predicted a non-repeated tone, compared to conditions in which the first tone validly predicted a repetition of the first tone. In other words, the effect of expectations on a hierarchically lower level as indexed by the difference in neural responses to repeated vs. alternated tones was dampened when expectations on a higher level as induced by the different transition probabilities were confirmed, as compared to situations in which these expectations were violated. Furthermore, Chennu et al. (2013) reported evidence that evoked potentials assumed to reflect the magnitude of prediction errors were reduced when instructions induced participants to track the respective deviations in order to report their number at the end of a block of trials. These observations converge upon the notion that validating prior expectations on a hierarchically higher level dampens prediction error signaling on a hierarchically lower level.

As already discussed in the Introduction, the Guessing and Choosing conditions of the present experiment did not only differ with respect to what participants were instructed to do (to guess vs. to choose) but also with respect to the amount and validity of information provided to them. This ambiguity was put up with in order to create a situation in which an identical proportion of (un)expected tasks could be implemented with both instructions. While Guessing and Choosing instructions surely impose different constraints regarding the proportion of trials conforming to participants’ guesses or choices, this does not mean that it would be impossible to independently vary the proportion of expected tasks within each of these conditions – albeit within certain limits that are surely different for both instructions. Thus, it may be worthwhile to tear apart these factors in future work.

Our findings may also bear some relevance with respect to the distinction between cued and voluntary task switching (cf. Arrington et al., 2014, for a review). As noted previously (Kleinsorge and Scheil, 2015), the Guessing condition may control and constrain processes of guessing the upcoming task that may proceed spontaneously in the cued variant of the task switching paradigm. The assumption that participants, even when not asked to do so, may try to anticipate the next task follows directly from the assumption that the brain is an intrinsically predictive device. Our new Choosing condition, on the other hand, approximates a voluntary task switching condition. Therefore, the procedure introduced in the present paper may offer an alternative route for investigating functional differences between cue-induced vs. self-generated expectations (cf. Gaschler et al., 2014).

In sum, the present study adds support to the assumption that errors in predicting an upcoming task result in an adaptation that reduces switch costs (cf. Kleinsorge and Scheil, 2015). The observation that this process is modulated by more abstract features of participants’ task representation adds support to the assumption that the corresponding processes are mediated top-down to support the exertion of cognitive control.

## Author Contributions

TK and JS designed the experiment. JS collected and analyzed the data. TK drafted the first version of the paper, TK and JS edited it and approved the final version.

## Conflict of Interest Statement

The authors declare that the research was conducted in the absence of any commercial or financial relationships that could be construed as a potential conflict of interest.
